# Synthesis and Characterization of Polystyrene-Supported Piperazine-Substituted Triazoles by CuAAC and First Evaluation for Metal Ion Extraction

**DOI:** 10.3390/polym8050187

**Published:** 2016-05-10

**Authors:** Riadh Slimi, Raja Ben Othman, Noomene Sleimi, Abid Ouerghui, Christian Girard

**Affiliations:** 1 Unité de Technologies Chimiques et Biologiques pour la Santé, CNRS UMR8258, INSERM U1022, Ecole Nationale Supérieure de Chimie de Paris, PSL Research University, 11 rue Pierre & Marie Curie, Paris 75005, France; riadhslimi82@yahoo.fr (R.S.); raja_both@yahoo.fr (R.B.O.); 2 Faculté des Sciences de Bizerte, Université de Carthage, Jarzouna, Bizerte 7021, Tunisia; noomene.sleimi@gmail.com; 3 Institut Supérieur de Biotechnologie, Avenue Habib Bourguiba, Beja 9000, Tunisia

**Keywords:** Tailor-made polymers, Merrifield resin, click chemistry, piperazine triazoles, metal ion removal

## Abstract

The goal of this work was to synthesize substituted polystyrene for metal extraction and/or depollution by introduction of substituted piperazines as chelatants starting from Merrifield polymer. After transformation of Merrifield’s resin in azidomethyl polystyrene, click-chemistry using copper (I)-catalyzed Huisgen’s reaction (CuAAC) was performed to prepare different polymers grafted with 1,4-triazoles bearing the piperazines, containing an alkyne as the other counterpart in the CuAAC. The polymers were then first tested for their efficiency to remove various metal ions from neutral aqueous solutions (Fe^3+^, Ni^2+^, Cu^2+^, Zn^2+^ and Pb^2+^). The polymers were found to extract Ni^2+^ and Zn^2+^ with low efficiencies ≤40%. For Fe^3+^ and Cu^2+^, the average extraction was around 80%, and for Pb^2+^ around 50%. The global selectivity for these polymers was found to be in the order of Fe^3+^ ≥ Cu^2+^ > Pb^2+^ >> Ni^2+^ > Zn^2+^.

## 1. Introduction

Water pollution by inorganic and organic contaminants is a major concern for water treatment and water stocks management on our planet. These contaminations often occur as a result of human agricultural and industrial activities, but can also be from natural origins, and can be found in all water reservoirs. Metallic ion pollution can have adverse effects on ecosystems and humans, mainly through the drinkable water quality. This kind of pollution is a major concern and solutions to prevent, monitor and treat it are still needed. Metallic cations can be removed from water using clays, natural and synthetic minerals (e.g., zeolites), ceramics and organic polymers bearing the required functions.

For a long time, what we call ion exchange resins, polymers bearing groups chelating a non-toxic metal cation such as sodium or others that can be exchanged with the cation for removal, have been used [1,2,3]. Several polymers can be made, and substitutions introduced, in order to create hydrophilic [4,5,6,7] and hydrophobic polymers [8,9], in order to use them in different applications [10,11,12,13,14]. Ideally, a polymer that can be easily functionalized for a specific application is of great interest. Such polymers can be engineered in a simple way to introduce chelating groups that can complex the metallic ions and find uses in trace detection, depollution or even supported catalysis [15,16,17,18,19,20].

Since our laboratory has been working on metal chelation for supported catalysts, we became interested in the preparation of engineered polymers [21,22,23]. We decided to introduce substituents on the polymer by using the “click-chemistry” concept and the best example of this approach, namely the copper (I)-catalyzed Huisgen's cycloaddition (“copper (I)-catalyzed azide/alkyne cycloaddition” or CuAAC) [24,25,26,27,28,29]. The use of CuAAC gives the opportunity to introduce different chelating groups on an azided polymer by selecting substituted alkynes [30,31,32,33,34,35].

**Figure 1:**
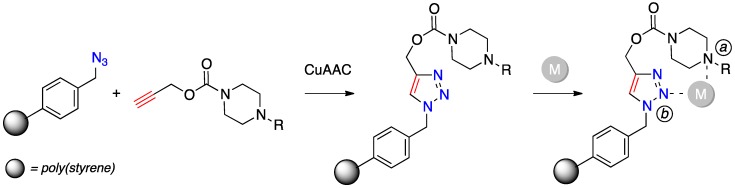
General scheme for the preparation of triazolic-piperazine polymers: Possible chelation methods as pendant (**a**), triazole (**b**) and integrated designs (**a**,**b**).

As depicted in Figure 1, the introduced appendages can chelate the metallic cation (“pendant design”, ***a***), but chelation can also occurs from the triazole linkage (“triazole design”, ***b***), or both of these methods (“integrated design”, ***a***,***b***). The chelation can also be made in mono- or multi-ligand mode, with other chelatants on the polymeric structure.

Our previous work was based on amide appendages [23], and we present in this paper the complete synthesis and characterizations of polymer-supported triazole-piperazines using Merrifield polymer (chloromethylated polystyrene) as the starting scaffold, chloride substitution to give azidomethyl polystyrene, followed by CuAAC with piperazine-substituted alkynes as carbamates of propargyl alcohol (Figure 1). The polymers were then first tested for their ability to extract some metallic cations (Fe^3+^, Ni^2+^, Cu^2+^, Zn^2+^ and Pb^2+^) from neutral aqueous solutions by atomic absorption spectrometry. The polymers synthesized here were also used in parallel to elaborate a high-throughput evaluation of chelation by conductometry, which has been published elsewhere [36].

## 2. Experimental

### 2.1. Materials

Merrifield polymer (chloromethylated poly-styrene-*co*-divinylbenzene (1%), 100–200 mesh, 2.1 mmol Cl·g^−1^) was purchased from Fluka / Sigma-Aldrich (St-Quentin Fallavier, France), washed alternatively three times with methanol and methylene chloride (10 mL·g^−1^) and dried before use. *N*-methylpiperazine (**1a**, ≥99%), sodium carbonate (≥98%), FeCl_3_·6H_2_O (97%), NiCl_2_·6H_2_O (98%), ZnSO_4_·7H_2_O (98%) and CuCl_2_·2H_2_O (97%) were issued from the same provider and used as such. Methylene chloride (99.5%), propargyl chloroformate (96%), *N*-ethyl (**1b**, 98%), 2-methoxyphenyl (**1c**, 98%), 2-pyrimidyl (**1d**, 98%), 2-pyridyl (**1e**, 98%), benzoyloxycarbonyl (**1f**, 98%) and 2-furoyl (**1g**, 97%) piperazines, and copper (I) iodide 99%) were provided by Sigma-Aldrich (St-Quentin Fallavier , France) and used as such. Sodium azide (99%) was supplied by Alfa-Aesar (Schiltigheim, France). Acetonitrile (99.5%), Pb(NO_3_)_2_ (99.5%) were ordered from SDS-Carlo-Erba and used as received. Methanol (97.8%), and triethylamine (99%) were bought from Prolabo/VWR (Fontenay sous Bois, France). Tetrahydrofuran (99%) was from the same source, but distilled from benzophenone sodium ketyl under nitrogen prior to use. Pyridine (99%) was ordered from Riedel-de Haën/Sigma-Aldrich (St-Quentin Fallavier, France) and used directly as furnished.

### 2.2. Organic Syntheses

#### 2.2.1. Syntheses of Azidomethyl Polystyrene (**A**) and Piperazine Propargyl Carbamates (**2a**–**2g**)

**Scheme 1:**
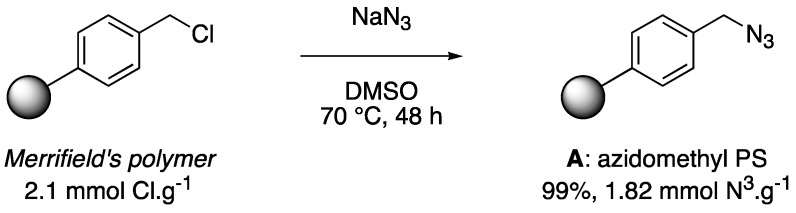
Preparation of azidomethyl poly-styrene (**A**) from Merrifield resin.

**Scheme 2:**
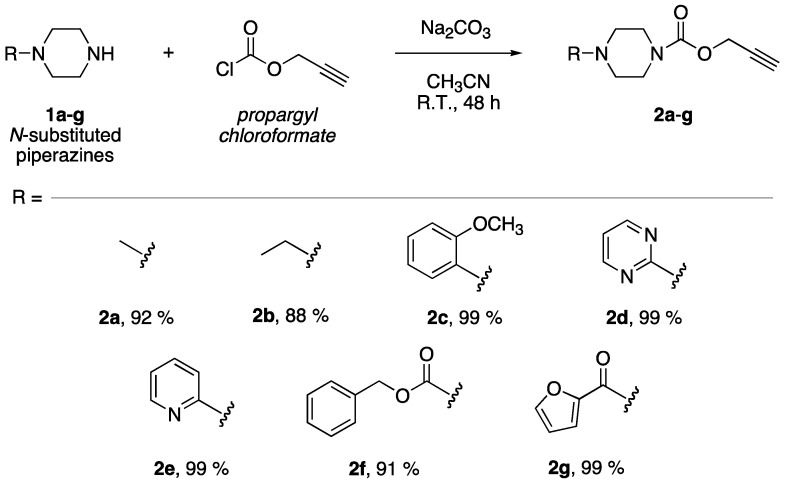
Synthesis of piperazine propargyl carbamates **2a**–**g** from *N*-substituted piperazine **1a**–**g** and propargyl chloroformate.

**Scheme 3:**
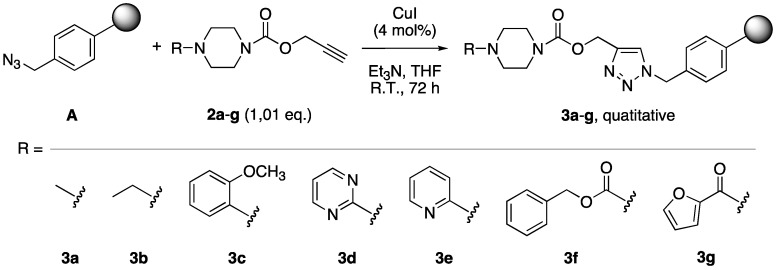
Cu(I)-catalyzed Huisgen's reaction of azidomethyl polystyrene (**A**) with piperazines propargyl carbamates **2a**–**g** to obtain the triazolic piperazines polymers **3a**–**g**.

Organic syntheses (Scheme 1, Scheme 2 and Scheme 3, Section 3.1 and Section 3.2) were conducted under nitrogen, otherwise stated, using standard methods and procedures. Characterizations were made as described below. Melting points (mp) were measured on a Kofler apparatus after calibration around the observed fusion of the product. Infrared analysis using the attenuated total reflectance technique (ATR/FTIR) was made on a Nicolet FTIR 200 spectrophotometer (Thermo Scientific, Villebon sur Yvette, France) between 4000 and 400 cm^−1^. Only the main and relevant absorption bands are indicated as vibrations (ν) and angular deformations (δ). Nuclear magnetic resonance spectroscopy (NMR) was recorded on a Bruker Avance 300 WB spectrometer (Bruker, Champs sur Marne, France) at 300 MHz for the proton spectra (^1^H) and 75.5 MHz for the carbon spectra (^13^C). Chemical shifts (δ) are indicated after calibration on the residual undeuterated solvent peak in parts per million (ppm) and as follow: s (singlet), d (doublet), t (triplet), dd (doublet of doublet), td (triplet of doublet ), m (multiplet). Coupling constants (*J*) are given in Hz. High performance liquid chromatography coupled with mass spectrometry (LC-MS) were performed on a Shimadzu LC-10AD VP chromatograph using an Alltima HP C8 3 µ reverse phase column (Alltech, Carquefou, France, length = 53 mm, internal diameter = 7 mm), using a acetonitrile/0.1% formic acid in water gradient at 1 mL·min^−1^ using the following sequence: 0–1 min, 30% CH_3_CN; 1–5 min 30% to 100% CH_3_CN; 5–12 min, 100% CH_3_CN; 12–15 min, 100% to 30% CH_3_CN and 15–20 min 30% CH_3_CN. Detection of the species was done using both PDA SPD-M10A VP (diode array, D_2_ lamp, 190–400 nm) and ELSD-LT (low temperature evaporative light scattering) detectors. Mass spectrometry was performed on a *prep* LCMS-2010A (liquid chromatograph mass spectrometer) at the end of the HPLC process between *m*/*z* = 100 to 1700 in positive mode by ESI with quadrupole at 5 V and detector at 1.5 kV. Results are given in ELSD purity, retention time (*R*_t_), and *m*/*z* of the ion.

##### Synthesis of Azidomethyl Polystyrene (**A**) (Scheme 1, Section 3.1)

The polymer **A** was prepared according to literature [37]. Merrifield polymer (2.10 mmol Cl·g^−1^, 5.00 g, 10.5 mmol Cl) was suspended in 50 mL DMSO and 5.00 g NaN_3_ (76.9 mmol, 7.32 eq.) were added. The reaction mixture was slowly magnetically stirred at 70 °C for 48 h. The cooled mixture was poured in water (50 mL), filtered on sintered glass and washed eight times with water (8 × 25 mL) and finally dried in an oven at 50 °C overnight. Elemental analysis of the isolated polymer gave 7.63% N and 0.10% Cl contents. Calculated substitution efficiency was 99% and the polymer has a final loading of 1.82 mmol N_3_·g^−1^.

ATR/FTIR: *ν* = 3036, 2934, 2864, 2103 (*ν*_N3_), 1605, 1507, 1451, 1256, 816, 760, 701, 547 cm^−1^.

##### Synthesis of Propargyl Carbamate Derivatives of Piperazine **2a**–**g** (Scheme 2, Section 3.1)

To a solution of the required *N*-substituted piperazine **1a** to **1g** (12.0 mmol) in acetonitrile (45 mL) was added Na_2_CO_3_ (1.27 g, 12.0 mmol, 1 eq.). Propargyl chloroformate (1.42 g, 1.17 mL, 12.0 mmol, 1 eq.) was then added dropwise. The reaction mixture was stirred for 48 h at room temperature and then filtered and evaporated under vacuum. The resulting carbamates **2a**–**g** were sufficiently pure to be used without further purification.

*Prop-2-yn-1-yl 4-methylpiperazine-1-carboxylate* (**2a**): C_9_H_14_N_2_O_2_. *M*_w_ = 182.21 g·mol^−1^. Yield = 92%. Oil. ATR/FTIR: ν = 3267, 2956, 2810, 1714, 1445, 1388, 1363, 1299, 1266, 1244, 1156, 1111, 1079, 769, 685, 599 cm^−1^. ^1^H NMR (300 MHz, CDCl_3_, δ): 2.28 (s, 3H), 2.36 (s, 4H), 2.45 (t, *J* = 2.41 Hz, 1H), 3.50 (t, *J* = 5.09 Hz, 4H), 4.68 (d, *J* = 2.44 Hz, 2H) ppm. ^13^C NMR (75.5 MHz, CDCl_3_, δ): 43.73, 46.09, 52.93, 54.58, 74.56, 78.44, 154.3 ppm. LC-MS: ELSD pur. 100%; *R*_t_ = 1.582 min; *m*/*z*: 183 ([M+H]^+^).

*Prop-2-yn-1-yl 4-ethylpiperazine-1-carboxylate* (**2b**): C_10_H_16_N_2_O_2_. *M*_w_ = 196.24 g·mol^−1^. Yield = 88%. Oil. ATR/FTIR: ν = 3264, 2987, 2828, 2782, 1715, 1445, 1388, 1360, 1289, 1249, 1137, 1086, 1034, 769, 684, 597 cm^−1^. ^1^H NMR (300 MHz, CDCl_3_, δ): 1.06 (t, *J* = 7.21 Hz, 3H), 2.4 (m, 7H), 3.50 (t, *J* = 5.11 Hz, 4H), 4.68 (d, *J* = 4.28 Hz, 2H) ppm. ^13^C NMR (75.5 MHz, CDCl_3_, δ): 11.91, 43.88, 52.34, 52.44, 52.94, 74.48, 78.53, 154.3 ppm. LC-MS: ELSD pur. 100%; *R*_t_ = 1.637 min; *m*/*z*: 197 ([M+H]^+^.

*Prop-2-yn-1-yl 4-(2-methoxyphenyl)piperazine-1-carboxylate* (**2c**): C_15_H_18_N_2_O_3_. *M*_w_ = 274.31 g·mol^−1^. Yield = 99%. mp = 105 °C. ATR/FTIR: ν = 3250, 3080, 3006, 2893, 2834, 1686, 1596, 1506, 1444, 1363, 1281, 1248, 1155, 1093, 1028, 988, 747, 587 cm^−1^. ^1^H NMR (300 MHz, CDCl_3_, δ): 2.48 (t, *J* = 1.83 Hz, 1H), 3.01 (s, 4H), 3.67 (t, *J* = 4.84 Hz, 4H), 3.86 (s, 3H), 4.72 (d, *J* = 1.83 Hz, 2H), 6.99 (m, 4H) ppm. ^13^C NMR (75.5 MHz, CDCl_3_, δ): 44.19, 50.53, 52.97, 55.44, 74.80, 78.60, 111.5, 118.5, 121.1, 123.5, 140.9, 152.3, 154.3 ppm. LC-MS: ELSD pur. 100%; *R*_t_ = 4.845 min; *m*/*z*: 275 ([M+H]^+^).

*Prop-2-yn-1-yl 4-(pyrimidin-2-yl)piperazine-1-carboxylate* (**2d**): C_12_H_14_N_4_O_2_. *M*_w_ = 246.26 g·mol^−1^. Yield = 99%. mp = 120 °C. ATR/FTIR: ν = 3270, 3043, 2942, 2884, 1726, 1596, 1514, 1448, 1551, 1284, 1241, 1131, 1095, 1022, 986 , 960, 795, 686, 498 cm^−1^. ^1^H NMR (300 MHz, CDCl_3_, δ): 2.51 (t, *J* = 2.45 Hz, 1H), 3.60 (t, *J* = 5.25 Hz, 4H), 3.86 (t, *J* = 5.06 Hz, 4H), 4.70 (d, *J* = 2.46 Hz, 2H), 6.55 (t, *J* = 4.76 Hz, 1H), 8.34 (d, *J* = 4.75 Hz, 2H) ppm. ^13^C NMR (75.5 MHz, CDCl_3_, δ): 43.44, 43.74, 53.15, 74.70, 78.42, 110.4, 154.5, 157.8, 161.5 ppm. LC-MS: ELSD pur. 100%; *R*_t_ = 4.251 min; *m*/*z*: 247 ([M+H]^+^).

*Prop-2-yn-1-yl 4-(pyridin-2-yl)piperazine-1-carboxylate* (**2e**): C_13_H_15_N_3_O_2_. *M*_w_ = 245.27 g·mol^−1^. Yield = 99%. mp = 92 °C. ATR/FTIR: ν = 3261, 2956, 2873, 1703, 1601, 1487, 1441, 1571, 1289, 1242, 1170, 1123, 1093, 984, 777, 742, 696, 584 cm^−1^. ^1^H NMR (300 MHz, CDCl_3_, δ): 2.52 (t, *J* = 2.44 Hz, 1H), 3.60 (m, 8H), 4.77 (d, *J* = 2.45 Hz, 2H), 6.69 (t, *J* = 6.06 Hz, 1H), 7.53 (td, *J* = 7.86 Hz, *J* = 2.09 Hz, 2H), 8.23 (dd, *J* = 4.76 Hz, *J* = 1.02 Hz, 1H) ppm. ^13^C NMR (75.5 MHz, CDCl_3_, δ): 43.60, 45.02, 53.09, 74.70, 78.44, 107.3, 113.8, 137.7, 148.2, 154.4, 159.1 ppm. LC-MS: ELSD pur. 100%; *R*_t_ = 1.808 min; *m*/*z*: 247 ([M+2H]^+^).

*Prop-2-yn-1-yl 4-(benzyloxycarbonyl)piperazine-1-carboxylate* (**2f**): C_16_H_18_N_2_O_4_. *M*_w_ = 302.32 g·mol^−1^. Yield = 91%. mp = 76 °C. ATR/FTIR: ν = 3248, 2953, 2914, 2882, 1697, 1507, 1438, 1360, 1291, 1238, 1110, 990, 952, 919, 868, 758, 591, 557 cm^−1^. ^1^H NMR (300 MHz, CDCl_3_, δ): 2.51 (t, *J* = 2.45 Hz, 1H), 3.49 (m, 8H), 4.71 (d, *J* = 2.46 Hz, 2H), 5.15 (s, 2H), 7.34–7.38 (m, 5H) ppm. ^13^C NMR (75.5 MHz, CDCl_3_, δ): 43.61, 53.19, 67.43, 74.89, 78.28, 128.0, 128.2, 128.6, 136.4, 154.3, 155.1 ppm. LC-MS: ELSD pur. 96%; *R*_t_ = 5.213 min; *m*/*z*: 343 ([M+MeCN]^+^).

*Prop-2-yn-1-yl 4-(furan-2-carbonyl)piperazine-1-carboxylate* (**2g**): C_13_H_14_N_2_O_4_. *M*_w_ = 262.26 g·mol^−1^. Yield = 99%. mp = 102 °C. ATR/FTIR: ν = 3238, 3129, 2947, 2889, 1718, 1631, 1589, 1497, 1432, 1355, 1286, 1255, 1191, 1123 ,1087, 1022, 769, 710, 598 cm^−1^. ^1^H NMR (300 MHz, CDCl_3_, δ): 2.51 (t, *J* = 2.45 Hz), 3.6 (m, 4H), 3.82 (t, *J* = 0.33 Hz, 4H), 4.74 (d, *J* = 2.45 Hz, 2H), 6.51(dd, *J* = 3.49 Hz, *J* = 1.78 Hz, 1H), 7.06 (dd, *J* = 3.49 Hz, *J* = 0.85Hz, 1H), 7.51 (dd, *J* = 1.78 Hz, *J* = 0.87 Hz, 1H) ppm. ^13^C NMR (75.5 MHz, CDCl_3_, δ): 54.64, 63.90, 86.18, 89.19, 122.2, 127.5, 154.9, 158.3, 164.9, 169.7 ppm. LC-MS: ELSD pur. 100%; *R*_t_ = 3.993 min; *m*/*z*: 263 ([M+H]^+^).

#### 2.2.2. Synthesis of Polymer-Supported Triazolic Piperazines **3a**–**g** (Scheme 3, Section 3.2)

Coupling reactions onto the polymer using CuAAC were conducted accordingly to the general procedure indicated below in round bottom flasks equipped with a reflux condenser. To a suspension of 3.00 g of azidomethyl polystyrene **A** (1.82 mmol N_3_·g^−1^, 5.46 mmol N_3_) in THF (60 mL) was added 6.30 mmol (1.15 eq.) of the alkyne (**2a**–**g**), 9.00 ml of triethylamine (6.75 g, 66.7 mmol, 12.2 eq.) and 2.40 mg of copper (I) iodide (12.6 µmol, 4 mol %). The suspension was slowly stirred at room temperature 72 h. After this time, the complete disappearance of the IR band of the azide of the polymer **A** (2103 cm^−1^) was observed. The resulting polymer was filtered on sintered glass and washed sequentially with CH_2_Cl_2_, pyridine, and MeOH (60 mL each), the sequential washings were repeated twice. The resulting polymers **3a**–**g** were finally dried overnight in an oven at 50 °C.

*1'-[(1',2',3'-triazol-4'-yl)methyl 4-methyl-piperazine-1-carboxylate]methyl polystyrene* (**3a**): Yellow powder. 1.50 mmol pipera-zine·g^−1^. ATR/FTIR: ν = 3039, 2942, 2872, 2809, 1704, 1621, 1511, 1441, 1380, 1298, 1236, 1153, 1112, 1055, 1008, 820, 767, 702, 551 cm^−1^.

*1'-[(1',2',3'-triazol-4'-yl)methyl 4-ethylpi-perazine-1-carboxylate]methyl polystyrene* (**3b**): Orange powder. 1.47 mmol piperazi-ne·g^−1^. ATR/FTIR: ν = 3038, 2938, 2872, 2827, 1705, 1613, 1507, 1442, 1382, 1289, 1249, 1134, 1060, 1028, 967, 820, 765, 702, 547 cm^−1^.

*1'-[(1',2',3'-triazol-4'-yl)methyl-4-(2-methoxyphenyl)piperazine-1-carboxylate]methyl polystyrene* (**3c**): Brown powder. 1.32 mmol piperazine·g^−1^. ATR/FTIR: ν = 3037, 2934, 2864, 1706, 1603, 1506, 1446, 1383, 1287, 1245, 1126, 1087, 1033, 946, 753, 702, 547 cm^−1^.

*1'-[(1',2',3'-triazol-4'-yl)methyl-4-(pyrimidin-2-yl)piperazine-1-carboxylate]methyl polystyrene* (**3d**): Yellow powder. 1.37 mmol piperazine·g^−1^. ATR/FTIR: ν = 3039, 2935, 2870, 1706, 1591, 1502, 1438, 1556, 1298, 1242, 1132, 1092, 1054, 981, 800, 761, 703, 555 cm^−1^.

*1'-[(1',2',3'-triazol-4'-yl)methyl-4-(pyridin-2-yl)piperazine-1-carboxylate]methyl poly-styrene* (**3e**): Brown powder. 1.37 mmol piperazine·g^−1^. ATR/FTIR: ν = 3037, 2938, 2864, 1705, 1601, 1485, 1437, 1382, 1285, 1235, 1171, 1128, 1053, 981, 837, 771, 703, 534 cm^−1^.

*1'-[(1',2',3'-triazol-4'-yl)methyl-4-(benzyl-oxycarbonyl)piperazine-1-carboxylate]methyl polystyrene* (**3f**): Pale pink powder. 1.27 mmol piperazine·g^−1^. ATR/FTIR: ν = 3039, 2934, 2869, 1706, 1613, 1507, 1429, 1365, 1293, 1231, 1101, 1056, 1016, 980, 830, 763, 703, 546 cm^−1^.

*1'-[(1',2',3'-triazol-4'-yl)methyl-4-(furan-2-carbonyl)piperazine-1-carboxylate]methyl polystyrene* (**3g**): Beige powder. 1.34 mmol piperazine·g^−1^. ATR/FTIR: ν = 3038, 2937, 2859, 1706, 1634, 1580, 1494, 1430, 1373, 1289, 1234, 1186, 1122, 1055, 1017, 843, 759, 703, 547 cm^−1^.

### 2.3. Characterizations by ATR/FTIR of Polymer-Supported Triazolic Piperazines

**Figure 2:**
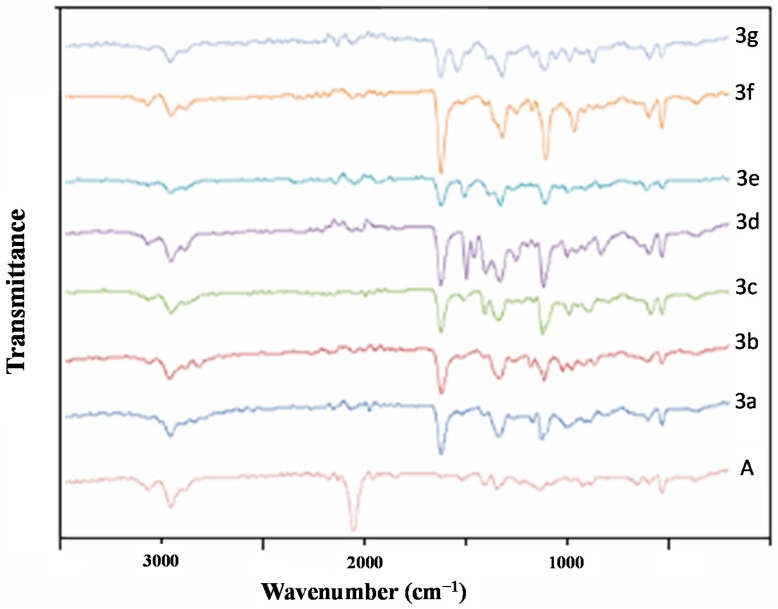
ATR/FTIR spectra of triazolic polymers **3a**–**g** and of the starting azidomethyl polystyrene (**A**).

Infrared analyses using the attenuated total reflectance technique (ATR/FTIR) were recorded as previously described for organic compounds (*vide supra*) Results appear in Figure 2, Section 3.1.

### 2.4. Metal Ion Extraction Method and Quantification

Neutral aqueous solutions of metal salts were prepared with 50 mg of FeCl_3_·6H_2_O (0.185 mmol), CuCl_2_·2H_2_O (0.293 mmol), NiCl_2_·6H_2_O (0.210 mmol), ZnSO_4_·7H_2_O (0.174 mmol), and Pb(NO_3_)_2_ (0.151 mmol) in 1 L of distilled water.

**Figure 3:**
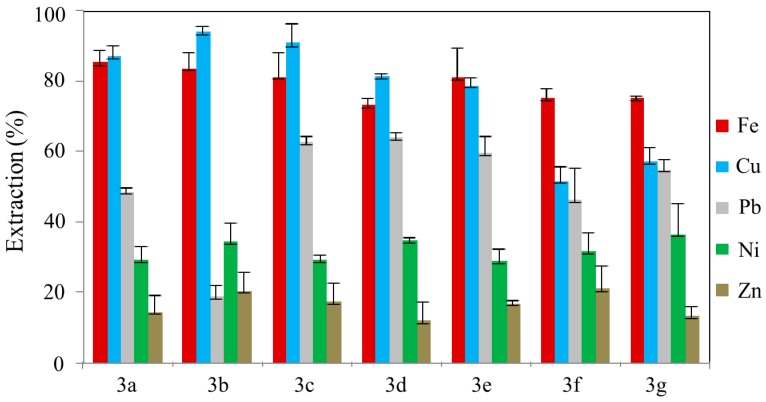
Extraction efficiencies of metal cations by the triazolic piperazine polymers **3a**–**g** (by polymer, in triplicate).

Aliquots of 250 mg of each polymer (**3a**: 0.342 mmol, **3b**: 0.335 mmol, **3c**: 0.302 mmol, **3d**: 0.315 mmol, **3e**: 0.315 mmol, **3f**: 0.292 mmol, **3g**: 0.307 mmol,) were incubated in triplicate with 20 mL (50 mg·L^−1^, 1 mg of salt) of each metal ion solution (3.70 µmol Fe^3+^, 5.87 µmol Cu^2+^, 4.21 µmol Ni^2+^, 3.48 µmol Zn^2+^, and 3.02 µmol Pb^2+^) at 25 °C for 24 h. The suspension was then filtered on distilled water prewashed filter paper. Evaluation of the chelated metal was done by concentration measurement by atomic absorption spectrometry on a Perkin Elmer type PinAAcle 900 T, after calibration curves recording for all metals. The results, average of three experiments, were expressed as percentages of extraction of the metal (Figure 3, Section 3.3).

## 3. Results and Discussion

### 3.1. Synthesis and Characterization of Azidomethyl Polystyrene (**A**) and Propargyl Carbamate Derivatives of Piperazine **2a**–**g**

Following the method of Mioskowski *et al.* [37], Merrifield’s polymer was treated with an excess of sodium azide (7.32 eq.) in DMSO at 70 °C for 48 h (Scheme 1). The azidomethyl polystyrene (**A**) prepared using this method was submitted to elemental analysis, which gave results of 7.63% for N and 0.10% for Cl. The substitution yield was of 99%, and the polymer has a final loading of 1.82 mmol N_3_·g^−1^. The presence of the azido group was confirmed by IR spectroscopy on which its strong characteristic absorption was seen at 2103 cm^−1^ *ν*_N3_).

The required coupling partners for **A** were prepared using commercially available *N*-substituted piperazines (**1a**–**g**) and reacting them with propargyl chloroformate in the presence of sodium carbonate in acetonitrile at room temperature for 48 h (Scheme 2). The corresponding propargyl carbamates **2a**–**g** were obtained in good yields (88%–99%), without the need of a purification step. Structures of the products **1a**–**d** were confirmed by their characteristic signals in NMR (*ca.* δ = 2.5 (triplet, ≡C–**H**) and 4.7 (doublet, OC**H_2_**C≡CH) ppm in ^1^H-NMR; 75/78 (**C**≡**C**) and 154 (**C**=O) ppm in ^13^C-NMR), IR spectroscopies (*ca.* 3250 (*ν*_≡C–H_) and 1700 (*ν*_C=O_) cm^−1^), as well as by LC-MS.

### 3.2. Synthesis and ATR/FTIR Analysis of Polymer-Supported Triazolic Piperazines **3a–g**

Piperazine propargyl carbamates **2a**–**g** (1.15 eq.) and azidomethyl polystyrene (**A**) were coupled together following a CuAAC procedure (4 mol % CuI in the presence of Et_3_N) in order to obtain the corresponding polymer-supported 1,2,3-triazoles-piperazines **3a**–**g** (Scheme 3). The reactions were conducted at room temperature for 72 h, after which time the azide vibration (ν_N3_ = 2103 cm^−1^) completely disappeared from the resulting polymers. FTIR spectra of the synthesized polymers **3a**–**g** did not show any residual vibration band from the azido group (ν_N__3_, *ca.* 2100 cm^−1^), as previously mentioned, in either of the alkynes (ν_≡C–H_, *ca.* 3250 cm^−1^), as shown in Figure 2. Characteristic vibration for the carbamate bonds (ν_C=O_, *ca.* 1700 cm^−1^), and other vibrations due to the triazole, the piperazine and the substituents, can be observed when compared to the spectrum of polymer **A**. The reactions were considered as quantitative based on ATR/FTIR analyses and gave polymers with triazole-piperazine loadings between 1.17 and 1.37 mmol piperazine·g^−1^ (Table 1).

### 3.3. Metal Ion Extractions by the Polymer-Supported Triazolic Piperazines **3a–g**

After 24 h incubation on a 250 mg scale (~0.3 mmol) of the polymers (see Table 1) in 20 mL of 50 mg·L^−1^ solutions of the salts (1 mg of salt, see Section 2.4 for details), the percentages of extraction for each metal were calculated by AAS, each experiment having been carried out in triplicate. The results for each polymer as a function of the metallic cations are presented in Figure 3.

Extraction results showed than Fe^3+^ is well extracted by all the synthesized polymers. The maximum levels of extraction for this metal were obtained with polymers **3a** (85.40% ± 3.01%) and **3b** (83.67% ± 3.87%).

Other extraction efficiencies were correct, with the minimum encountered with **3d** (73.14% ± 1.53%), being in the range of 75%–81%, in the following decreasing order: **3e** (81.16% ± 8.02%), **3c** (81.29% ± 6.26%), **3g** (75.33% ± 0.19%), and **3f** (75.20% ± 2.38%). The average iron (III) extraction for this series of polymers was around 79%

The second well-extracted metal ion was copper (II), with some higher values than those for Fe^3+^. Once again, the best extractions were found with polymers **3a** (87.27% ± 2.52%) and **3b** (94.00% ± 1.22%), together with **3c** (90.79% ± 5.27%). The efficiencies for Cu^2+^ removal were then separated in two groups, with **3d** (81.64% ± 0.05%) and **3e** (78.94% ± 1.61%) near the first ones, then **3g** (57.37% ± 3.63%) and **3f** (51.80% ± 3.32%) being at a medium level. Average extraction for the polymeric series for Cu^2+^ was near 77%.

For Pb^2+^ capture from the aqueous solutions by these piperazine-triazolic polymers, it did not follow exactly the same order as for the two previous metals, with an average extraction in the range of 50%. There was an obvious difference between **3a** (48.53% ± 0.71%) efficiency being in the average range, and the strong loss for **3b** (18.93% ± 2.73%), being the less powerful extracting polymer. Poly(styrenes) **3d** (64.27% ± 0.81%), **3c** (62.68% ± 1.17%) and **3e** (59.67 ± 4.26%) were more or less 10% above the average, and **3g** (54.89% ± 2.44%) and **3f** (46.28% ± 8.71%) around it.

For Ni^2+^, extraction efficiencies were in a narrow distribution around an average value of 32%. None of the studied polymers seemed to retain more of this metallic ion. The results were the following: **3a** (29.37% ± 3.20%), **3b** (34.68% ± 4.59%), **3c** (29.28% ± 1.06%), **3d** (34.98% ± 0.17%), **3e** (28.91% ± 2.89%), **3f** (31.87% ± 5.57%) and **3g** (36.44% ± 8.25%).

Finally, the least-extracted metal was zinc (II). As for Ni^2+^, none of the polymers was much better than the low average of 16%. Polymers **3a** (14.53% ± 4.20%), **3b** (20.45% ± 4.80%), **3c** (17.55% ± 4.58%), **3d** (12.01% ± 5.09%), **3e** (16.97% ± 0.38%), **3f** (21.01% ± 6.17%) and **3g** (13.34% ± 2.39%) showed a distribution again not very far from the calculated average.

When looking at all the results, as an average of all polymers, we can observe that the metals that are the best extracted are iron (III) and copper (II), at around 80%, lead (II) is extracted at around 50%, and nickel (II) and zinc (II) at 15%–30%. These results suggest that, by selecting the right polymer, a discrimination and selectivity for at least iron (III) and copper (II), and maybe lead (II), can be done over nickel (II) and zinc (II).

Since the polymeric structure differs only by the R substituent on the nitrogen (Scheme 3), a tentative analysis has been done to try to understand the influence of the substituent’s nature on the extraction efficiencies. Polymers **3a** and **3b** have an alkyl group on the terminal nitrogen, and are thus amines. For **3c**–**e**, and aromatic ring is the substituent, and the compounds are aniline derivatives.

Finally, for **3f** and **3g**, they include carbonyl linkages, and are in the carbamate and amide families, respectively. Considering only the R substituent, the availability of the nitrogen electron doublet for chelation should theoretically be in the following order: **3a**,**b** > **3c–e** > **3f**,**g**. It should be noted, however, that polymer **3e** also includes a pyridyl group, capable of metal chelation.

For iron (III), the nature of the *N*-terminal substituent of the piperazine does not seem to have a determinant effect on the chelation of this metal. Extraction efficiencies are within the same range of 70%–85%. Only a small decrease in efficiency can be observed in the previously cited order (**3a**,**b** > **3c–e** > **3f**,**g**), which may not be that significant.

In the case of Cu^2+^, a more obvious effect seems to take place, especially for carbamate (**3f**) and amide (**3g**) containing *N*-substituents. Polymers **3a** and **b** are a little more efficient (*ca.* 90%) than **3c**–**e** (*ca.* 80%), but there is a more obvious decrease of around 30% while using polymers **3f** and **3g**.

When looking at lead (II) extraction, the effects are more puzzling. *N*-alkyl-substituted polymers **3a** and **3b** are not as efficient as was the case for the two previous metallic ions, with ~50% and ~20% extraction, respectively. Aryl-substituted polymers **3c–e** are better (60%), while **3f** and **3g** are going down to the **3a** level.

For Ni^2+^and Zn^2+^, none of the polymers extracted them well. No conclusion can be drawn other than that these polymers are not suitable for their extraction.

The electro-negativities of the substituted nitrogen of the piperazine may partially explain some of the relative extraction efficiencies, at least for iron (III) and copper (II). For lead (II), the alkyl series (**3a**,**b**) is not really matching the previsions, while the other series do. For Ni^2+^ and Zn^2+^, none of the results can give an idea about this influence since they are not well extracted, and the levels are the same for all polymers studied here. These results may indicate that the awaited “pendant design” is not the major mode of complexation encountered in this series. It may be, in part, done by an integrated mode, or it may mainly be driven by the triazole one (Figure 1). Iron (III) and copper (II) are known to be “nitrogen-philic” metals, especially for trinitrogen-/azide-type species. In a previous work, such an effect has been observed [21,22,23].

Another influence of these terminal *N*-substituents can be the hydrophilic/hydrophobic balance of the polymers. This may explain the access and/or stability of complexes formed between the metal the triazole nucleus. This effect has already been observed in the case of copper (II) and lead (II) in a work on PVC-supported triazolic amides [38].

We have also tried to rationalize the interactions between the polymers and the metal ions based on their electropositivities, ionic radii and water solvation. The extraction efficiencies, based on the metal cation size or electro-positivities, cannot be taken into account since only lead (II) has a superior size and lower electro-positivity when compared to the transition metals having similar radii.

When studying the water solvation of the different metals used here, maybe some explanation can be found when trying to link metal and polymer properties. In Table 2, these are presented in decreasing order of removal of metallic cations. The pH of the initial solution of the cation (pH_i_) and the average pH after extraction by all polymers (pH_f_) are reported. We can observe that the pH of the solutions were between 3.2 and 7.4, and that there is no direct link between them and the extraction order. The pH at which the extraction is performed is important since a speciation can already be performed as a function of the metal ion [39]. In these cases, a “pH_50_”, a pH at which 50% of the cation is extracted, can be calculated. The pH_50_ values can be correlated in a linear way with another value, the first hydrolysis constant in water (pK_1_), related to the reaction M(OH_2_)_m_^n+^ = M(OH_2_)_m−1_(OH)^(n−1)+^ + H^+^ [39,40]. When looking at pK_1_, it can be observed that when the value increases, the extraction of the cation decreases almost accordingly. pH_50_ is very dependent on the substrate used for cation removal, but always is in linear relation with pK_1_ as previously mentioned. From published studies, the order of extraction and pH_50_/pK_1_ values increases in the order of Cu ≥ Pb >> Zn > Ni. From our results, it seems that for Fe^3+^, Cu^2+^ and Pb^2+^, the solutions as a function of the polymers are between pH_50_ and pH_90_. For Ni^2+^ and Zn^2+^, the extractions were never excellent, maybe being in a zone between pH_10_ and pH_40_. These results thus give the following order of extraction: Fe^3+^ ≥ Cu^2+^ > Pb^2+^ >> Ni^2+^ > Zn^2+^.

All the results and analyses cannot definitively identify the discrete complexation behavior of the metal by the polymers at the solid/liquid interface. However, we were able to obtain results that begin to give a better understanding of the subtle equilibrium between all factors influencing the extraction efficiencies of such polymers.

## 4. Conclusions

In this work, we studied the chemical grafting of piperazine-chelating units onto commercial poly[styrene] (Merrifield’s resin) using the CuAAC procedure between the azided polymer and selected piperazine-*N*-propargyl carbamates. The synthesized polymers were characterized by FTIR. They were then tested for their efficiency to extract metallic ions from aqueous solution, including Fe^3+^, Ni^2+^, Cu^2+^, Zn^2+^ and Pb^2+^. All polymers were found to extract Fe^3+^ and Cu^2+^ well.

Most of the polymers were found to have a good extraction potential for Pb^2+^. None of the polymers were able to extract Ni^2+^ and Zn^2+^ with good efficiencies, thus showing certain selectivity when compared to the other studied metallic ions. As an added proof, the extraction efficiencies were studied on 13 metallic cations by a “high-throughput” approach using conductometry [36]. Even if the method was less precise and sensitive, similar average affinities were found for Fe^3+^, Ni^2+^ and Pb^2+^ (13%–17% lower).

In this series, we obtained far better extraction results than with our first series of triazolic polymers, based on poly(styrene) and poly(vinyl chloride) backbones, bearing propargyl amides and propiolic anilides [23,38].

Even if no clear interpretation can be done with the results for the interfacial chelation process, the good extraction properties encourage us to continue polymer modifications using CuAAC in order to find new triazole-linked polymeric complexing agents for depollution, trace detection and catalytic applications [41,42,43,44,45,46,47]. Further studies will be reported in due course.

## Figures and Tables

**Table 1:** Masses, substitution and number of millimoles for 250 mg (incubation scale) of the synthesized polymer-supported triazolic piperazines **3a**–**g**.

Polymer ^a^	Mass increase (g) ^b^	Total mass (g)	Substitution (mmol·g^−1^) ^b^	250 mg (mmol)
**3a**	0.99	3.99	1.37	0.342
**3b**	1.07	4.07	1.34	0.335
**3c**	1.50	4.50	1.21	0.302
**3d**	1.34	4.34	1.26	0.315
**3e**	1.34	4.34	1.26	0.315
**3f**	1.65	4.65	1.17	0.292
**3g**	1.43	4.43	1.23	0.307

^a^ For structures, see Scheme 3; ^b^ Based on a quantitative transformation starting from 3 g (5.46 mmol N_3_) of polymer **A**.

**Table 2:** Average pH before and after incubation with polymers **3a**–**g**, and pK_1_ for each metal ion solution.

Cation	Anion	pH_i_^a^	pH_f_^b^	pK_1_^c^
Fe^3+^	Cl^−^	3.71 ± 0.01	3.19 ± 0.02	2.2
Cu^2+^	SO_4_^2−^	6.90 ± 0.01	7.06 ± 0.61	7.6
Pb^2+^	NO_3_^−^	5.07 ± 0.01	5.26 ± 0.59	7.7
Ni^2+^	Cl^−^	6.13 ± 0.01	7.36 ± 0.62	9.9
Zn^2+^	SO_4_^2−^	6.64 ± 0.01	5.71 ± 0.29	9.5

^a^ pH of the salt solution before incubation. Water pH was 6.66; ^b^ pH of the salt solution after incubation; ^c^ pK_1_ values from references included in [40].
